# Interaction between age and blood urea nitrogen to creatinine ratio on mortality in patients with severe cirrhosis: a retrospective cohort study from the MIMIC database

**DOI:** 10.3389/fendo.2025.1544223

**Published:** 2025-03-05

**Authors:** Yu Yi, Lin Li, Yinghua Chen, Yawen Luo

**Affiliations:** ^1^ Department of Infectious Diseases, The Affiliated Hospital of Zunyi Medical University, Zunyi, Guizhou, China; ^2^ Department of Oncology, Zibo Municipal Hospital, Zibo, China

**Keywords:** cirrhosis, mortality, blood urea nitrogen to creatinine ratio (BCR), outcome prediction, severely ill patients

## Abstract

**Background:**

Cirrhosis is a leading cause of global disease burden, with high mortality, particularly in critically ill patients. The blood urea nitrogen to creatinine ratio (BCR) is a straightforward biochemical indicator of renal excretory function and is linked to negative outcomes across different conditions. However, the relationship between BCR and mortality in critically ill patients with cirrhosis is unclear, The purpose of this study is to explore this question.

**Methods:**

A retrospective cohort study was performed utilizing the MIMIC-IV database. We divided BCR into quartiles and evaluated 180-day and 365-day mortality as the primary outcomes. Kaplan-Meier survival analysis and multivariate Cox regression modeling were used to assess the link between BCR and mortality. Linear relationships were further determined using restricted cubic spline (RCS) curves, and finally, subgroup analyses were also performed.

**Results:**

In our study of 2,816 critically ill cirrhotic patients, elevated BCR was significantly linked to higher mortality at both 180 and 365 days. The top BCR quartile showed a 45% higher risk of 180-day mortality (HR=1.45, 95% CI: 1.21-1.73) and a 38% higher risk of 365-day mortality (HR=1.38, 95% CI: 1.17-1.63) relative to the bottom quartile. RCS analysis demonstrated a notable linear correlation between BCR and mortality risk. Subgroup analyses indicated a stronger association between BCR and mortality among older patients.

**Conclusion:**

In critically ill cirrhotic patients, elevated BCR values are strongly linked to increased mortality risk. Our research highlights BCR’s potential as a prognostic marker for cirrhosis, especially in elderly patients.

## Introduction

Cirrhosis denotes the irreversible alterations in liver structure and function resulting from chronic damage, typically manifesting as hepatocyte necrosis, fibrosis, and nodule formation (1). Liver disease is responsible for 2 million deaths globally each year, accounting for 4 percent of all deaths, which are mainly attributable to complications of cirrhosis and hepatocellular carcinoma, cirrhosis is ranked as the 11th leading cause of death (2, 3). Cirrhosis-related mortality declined from 20.0/100,000 person-years in 1980 to 15.8/100,000 person-years in 2010 (4). Mortality has declined markedly in East Asia, North Africa/Middle East and high-income Asia, and the Pacific, but has also increased in many other parts of the world, including South Asia, Central Asia, and Eastern Europe (5). In addition, cirrhosis generates a significant number of disability-adjusted life-years (DALYs) and is the 15th leading cause of DALYs globally (3). Cirrhosis mortality is affected by factors like alcohol consumption, viral hepatitis (e.g., hepatitis B and C), and non-alcoholic fatty liver disease (6). Cirrhosis progresses from the asymptomatic phase (compensated cirrhosis) to the symptomatic phase (decompensated cirrhosis). Decompensated cirrhosis refers to a state where the liver function is no longer able to meet the body’s demands, with significant manifestations of liver failure, such as ascites, hepatic encephalopathy, or variceal bleeding (1). Patients with critically ill cirrhosis are usually admitted to the Intensive Care Unit (ICU) due to severe complications of cirrhosis (7). These patients frequently encounter complications such as multi-organ failure, infections, and bleeding, which exacerbate their condition and heighten the risk of mortality (8). Nearly 40 percent of patients with cirrhosis develop infections during admission or hospitalization. The most common types of infections included spontaneous bacterial peritonitis (22.5-25%), urinary tract infections (21.4-28.5%), respiratory tract infections (9.9-16.4%), skin and soft tissue infections (8.5-12.2%), and secondary bacterial peritonitis (4%), and the presence of any infection in patients with cirrhosis was associated with a 4-fold increase in mortality (3). Recent studies suggest that the prognosis of cirrhotic patients is closely associated with clinical parameters such as the Child-Pugh score, model for end-stage liver disease (MELD) score, liver function status, comorbidities, and infection severity (5, 9). Subsequent studies have built on this foundation by further refining the score to include additional biomarkers with independent predictive value, including the United Kingdom Model for End-Stage Liver Disease (UKELD) incorporating serum sodium, the MELD-Plus score incorporates albumin, total cholesterol, age, and length of hospital stay (10, 11). The MELD-EEG adds an electroencephalogram (EEG), which responds to the presence of hepatic encephalopathy, further increasing predictive accuracy (12). In addition, different strata have been created. For different strata, such as patients with acute or chronic liver failure (ACLF), the European Foundation for the Study of Chronic Liver Failure (CLIF) developed the CLIF Organ Failure (CLIF-OF) score (13, 14). In addition to the above composite scores, an independent index of heart rate variability has also been shown to be associated with cirrhosis and independently predicted mortality in cirrhotic patients (15). In conclusion, the identification of more biomarkers with independent predictive value is important for the prognostic management of cirrhosis.

The blood urea nitrogen to creatinine ratio (BCR) is a simple biochemical indicator of the kidney’s ability to excrete urea and creatinine (16). Recent studies have shown a strong link between high BCR levels and negative clinical outcomes in various patient groups, such as those with acute kidney injury, acute decompensated heart failure, chronic heart failure, and acute myocardial infarction (17). In the context of chronic heart failure, an increased BCR has been linked to heightened mortality (18). Moreover, the potential of the BCR as a prognostic marker for stroke is underlined by a large sample study showing a significant 19% increase in stroke risk in participants in the lowest quintile compared with those in the third quintile of the BCR (19). Acute kidney injury (AKI) is a prevalent comorbidity among cirrhotic patients, impacting up to 50% of hospitalized cirrhotic patients and 58% of ICU patients (20). Renal insufficiency occurs in about 20% of patients with cirrhosis, and fluctuations in creatinine in these patients are closely related to hepatic and renal impairment, and an elevated BCR may imply reduced hepatic metabolic function and impaired renal perfusion (21). Previous studies have shown that BCR is associated with short-term mortality in patients with cirrhosis and is a better predictor of mortality than MELD in patients with cirrhosis, but there is currently a lack of clarity regarding the association between different levels of BCR and mortality in patients with severe cirrhosis (22, 23).

We propose that a high BCR correlates with negative inpatient clinical outcomes in critically ill liver cirrhosis patients. This study assesses the link between BCR and overall mortality in critically ill liver cirrhosis patients using data from the Medical Information Mart for Intensive Care (MIMIC) database data, exploring BCR’s potential as a prognostic biomarker.

## Materials and methods

### Data sources

Our retrospective cohort study utilized the publicly accessible MIMIC-IV (version 2.2) database, comprising de-identified health data of patients admitted to the intensive care unit at Beth Israel Deaconess Medical Center from 2008 to 2019 (24). Lin Li, an author of the paper, was granted access to the database (Record No: 66829958). The MIMIC-IV database usage received approval from the review boards of both the Massachusetts Institute of Technology and Beth Israel Deaconess Medical Center. Informed consent was not required due to the anonymization of patient health information in the database (25). Our study adhered to the ethical principles of the Declaration of Helsinki.

### Study participants

**Figure 1:**
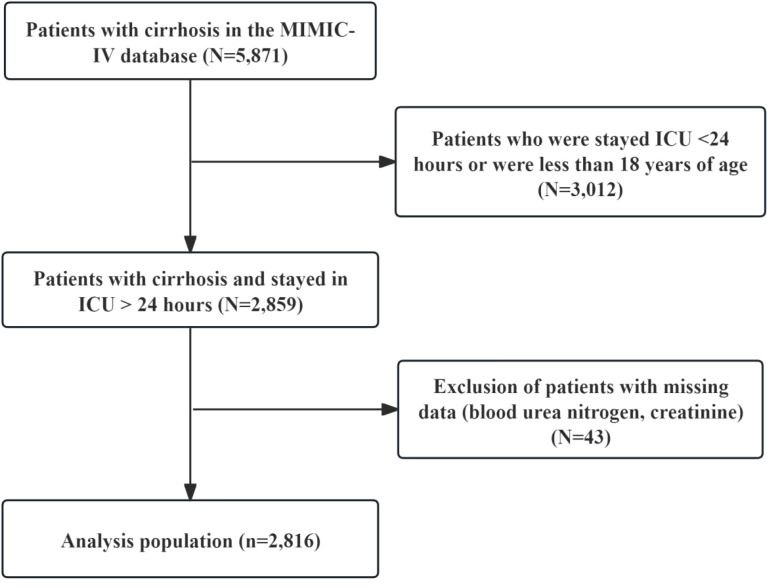
The flow chart of participants.

The diagnosis of cirrhosis relies on the International Classification of Diseases, Ninth Edition (ICD-9), including 5715, 5712, 5716, and Tenth Edition (ICD-10) codes, including k7469, k7460, k7031, k7030, and k743. The study initially enrolled 5,871 cirrhosis patients, excluding those with ICU stays under 24 hours, under 18 years of age, or lacking blood urea nitrogen or creatinine data. A total of 2,816 patients participated in this study (**Figure 1**).

Follow-up began at the time of ICU admission and continued until one year after the patient was discharged from the hospital or at the end of death. Patients who were alive as of the end of the study period were defined as right censored.

### Data extraction

Baseline characteristics related to cirrhosis, such as demographics, vital signs, lab results, comorbidities, and disease severity scores, were extracted using SQL scripts from the GitHub repository (https://github.com/MIT-LCP/mimic-iv) (26). The study considers demographic factors such as age and gender; Vital sign variables including weight, heart rate, systolic blood pressure (SBP), diastolic blood pressure (DBP), mean blood pressure (MBP), respiratory rate, transcutaneous arterial oxygen saturation (SpO_2_), and temperature; Laboratory results including white blood cell (WBC), red blood cell (RBC), platelets, hemoglobin, RDW, albumin, sodium, potassium, calcium, chloride, glucose, anion gap, international normalized ratio (INR), prothrombin time (PT) and partial thromboplastin time (PTT), alanine aminotransferase (ALT), and aspartate aminotransferase (AST); Comorbidities include atrial fibrillation, respiratory failure, acute kidney injury (AKI), hypertension, diabetes mellitus (DM), heart failure, myocardial infarction, malignant tumors, and liver transplantation; and disease severity scores such as sequential organ failure assessment score (SOFA), acute physiology score III (APS III), systemic inflammatory response syndrome score (SIRS), simplified acute physiology score II (SAPS II), and oxford acute severity of illness score (OASIS). All laboratory variables were derived from the first measurements of the patients at the time of admission.

MissForest interpolates better than established interpolation methods for data with missing values in the range of 10-30% (27). We chose to exclude variables with more than 20% missing data based on existing studies, while variables with less than 20% missing data were estimated using the Random Forest method in the R software missForest package (27, 28).

### Exposure and outcomes

The main exposure variable in this analysis was BCR, treated as a continuous variable and divided into four categories according to its quartiles: Q1 ≤ 14, 14<Q2 ≤ 20, 20<Q3 ≤ 27, and Q4>27 (16). Patient mortality at 180 and 365 days was the primary outcome of this study.

### Statistical analysis

Participant baseline characteristics are categorized by BCR quartiles. The Kolmogorov-Smirnov test was utilized to evaluate the normality of the variables. Normally distributed continuous variables are expressed as mean ± standard error, whereas skewed continuous variables are expressed as median with interquartile range (IQR). Categorical variables are expressed as frequencies (%). Continuous variables between groups were compared using one-way ANOVA or the Mann-Whitney U test, based on distribution normality. Categorical data comparisons utilized chi-square or Fisher’s exact tests, depending on suitability.

We employed Kaplan-Meier (KM) analysis and multivariate Cox regression modeling to evaluate the relationship between BCR and mortality in cirrhosis patients. To ensure the validity of the Cox regression model, we tested the proportional hazards assumption, the results are presented in **Supplementary Figure 1**. We developed three models: Model 1 is unadjusted; Model 2 is adjusted for age and body weight; and Model 3 was further adjusted for variables that differed in the baseline datasheet including WBC, RDW, hemoglobin, potassium, chloride, glucose, anion gap, INR, AST, SOFA, APS III, SIRS, heart rate, AKI, diabetes, sepsis and liver transplantation. To explore the potential linear relationship between BCR and mortality in cirrhosis patients, we applied restricted cubic spline (RCS) regression, adjusting for variables in Model 3. Subgroup analyses evaluated the consistency between BCR and mortality among patients with cirrhosis. These analyses were categorized by age, sex, presence of AKI, hypertension, diabetes, heart failure, and myocardial infarction. Interaction effects between subgroups were further statistically assessed.

All analyses were performed using R software (version 4.2.2), and a two-sided P value of less than 0.05 was considered statistically significant.

## Results

### Initial data on critically ill cirrhosis patients

The study categorized severe hepatic cirrhosis patients into four groups (Q1 to Q4) according to BCR quartiles and examined the baseline characteristic differences among these groups (**Table 1**). The results showed a significant difference in the age distribution of patients with increasing BCR, with the proportion of patients aged ≥60 years increasing and then decreasing, and being highest in group Q3 (P<0.05). Mean body weight showed a trend of increasing and then decreasing from Q1 to Q4 and was highest in group Q3 (P<0.05). In terms of laboratory parameters, leukocytes, potassium, chloride, and glucose increased with increasing BCR, while anion gap and AST showed an opposite trend (P<0.05). In addition, erythrocytes and hemoglobin first increased and then decreased with increasing BCR levels, while PTT and INR showed opposite trends. In terms of disease severity scores, SOFA, APSIII, SIRS, and OASIS first decreased and then increased with increasing BCR. Regarding vital signs, the heart rate initially decreased before increasing with the rise in BCR. In terms of complications, respiratory failure, AKI, and DM changed significantly with increasing BCR (all P values <0.05). Regarding patient prognostic indicators, patients with the highest BCR levels had the highest all-cause mortality at 180 and 365 days.

**Table 1:** Baseline characteristics of the cirrhosis population according to BCR quartiles.

	Total (n=2816)	Q1 (n=717)	Q2 (n=799)	Q3 (n=599)	Q4 (n=701)	*P*
Age, years						<0.001
<60	1377 (48.90)	425 (59.27)	393 (49.19)	250 (41.74)	309 (44.08)	
≥60	1439 (51.10)	292 (40.73)	406 (50.81)	349 (58.26)	392 (55.92)	
Gender, n (%)						0.342
female	958 (34.02)	236 (32.91)	268 (33.54)	196 (32.72)	258 (36.80)	
male	1858 (65.98)	481 (67.09)	531 (66.46)	403 (67.28)	443 (63.20)	
Weight, kg	85.72 ± 23.32	86.12 ± 23.36	86.65 ± 23.28	87.07 ± 23.73	83.10 ± 22.80	<0.001
WBC, K/µL	10.83 ± 7.61	10.19 ± 6.93	10.58 ± 7.61	10.81 ± 7.59	11.81 ± 8.20	<0.001
RBC, K/µL	3.12 ± 0.73	3.15 ± 0.73	3.19 ± 0.74	3.12 ± 0.70	3.00 ± 0.72	<0.001
Platelet, K/µL	124.80 ± 84.79	125.59 ± 86.23	127.77 ± 87.48	122.95 ± 75.62	122.18 ± 87.61	0.573
Hemoglobin, g/dL	9.78 ± 2.12	9.87 ± 2.15	10.00 ± 2.20	9.79 ± 1.97	9.42 ± 2.08	<0.001
RDW, %	16.95 ± 2.82	16.76 ± 2.80	16.53 ± 2.71	16.90 ± 2.74	17.66 ± 2.88	<0.001
Albumin, g/dL	2.98 ± 0.62	2.99 ± 0.66	3.01 ± 0.57	2.98 ± 0.57	2.95 ± 0.65	0.357
Sodium, mmol/L	136.58 ± 6.31	136.62 ± 6.30	136.73 ± 5.99	136.22 ± 6.44	136.68 ± 6.57	0.461
Potassium, mmol/L	4.28 ± 0.88	4.14 ± 0.88	4.23 ± 0.87	4.36 ± 0.83	4.40 ± 0.88	<0.001
Calcium, mg/dL	8.25 ± 1.05	8.18 ± 1.14	8.26 ± 0.97	8.27 ± 1.01	8.29 ± 1.08	0.180
Chloride, mmol/L	102.65 ± 7.47	101.44 ± 7.56	102.98 ± 7.17	102.98 ± 7.08	103.24 ± 7.90	<0.001
Glucose, mg/dL	141.82 ± 73.96	134.66 ± 71.83	141.98 ± 81.23	144.80 ± 68.46	146.43 ± 71.50	0.015
Anion gap, mmol/L	15.81 ± 5.65	17.44 ± 6.74	15.76 ± 5.64	15.24 ± 4.76	14.67 ± 4.68	<0.001
PT, sec	19.78 ± 9.13	20.37 ± 11.46	19.21 ± 8.21	19.56 ± 8.78	20.01 ± 7.55	0.077
PTT, sec	43.04 ± 21.70	44.64 ± 22.53	42.91 ± 21.70	43.21 ± 22.42	41.41 ± 20.07	0.048
INR	1.82 ± 0.84	1.88 ± 1.08	1.77 ± 0.78	1.79 ± 0.67	1.85 ± 0.74	0.040
ALT, u/dL	130.01 ± 359.46	143.60 ± 396.95	130.65 ± 373.03	138.98 ± 353.58	107.73 ± 303.75	0.252
AST, u/dL	283.54 ± 1059.02	401.69 ± 1578.02	277.30 ± 1032.55	252.27 ± 662.27	196.52 ± 591.76	<0.001
SOFA	7.80 ± 4.18	8.51 ± 4.61	7.46 ± 4.15	7.61 ± 3.93	7.63 ± 3.86	<0.001
APS III	55.91 ± 24.28	57.77 ± 27.33	52.40 ± 24.23	55.68 ± 23.30	58.19 ± 21.22	<0.001
SIRS	2.54 ± 0.93	2.64 ± 0.94	2.51 ± 0.93	2.49 ± 0.92	2.53 ± 0.94	0.015
SAPS II	39.38 ± 14.96	39.33 ± 15.88	37.92 ± 15.02	39.61 ± 14.22	40.91 ± 14.41	<0.010
OASIS	32.34 ± 8.75	32.86 ± 9.61	31.77 ± 8.61	32.38 ± 8.37	32.42 ± 8.28	0.115
Heart rate, beats/minute	90.68 ± 20.01	93.23 ± 20.99	89.13 ± 19.22	89.47 ± 19.63	90.88 ± 19.96	<0.001
SBP, mmHg	119.82 ± 23.92	121.04 ± 25.52	120.37 ± 23.25	118.66 ± 24.07	118.93 ± 22.79	0.119
DBP, mmHg	70.13 ± 99.91	71.06 ± 34.61	68.81 ± 19.85	75.66 ± 211.27	65.98 ± 17.02	0.355
MBP, mmHg	82.81 ± 115.25	91.10 ± 226.17	81.31 ± 18.86	79.30 ± 18.93	79.04 ± 16.70	0.161
Respiratory rate, breath/minute	19.33 ± 6.33	19.56 ± 6.33	18.97 ± 6.11	19.24 ± 7.07	19.59 ± 5.89	0.187
SpO2, %	97.11 ± 16.98	96.59 ± 5.91	97.83 ± 31.05	97.02 ± 3.62	96.88 ± 3.51	0.517
Temperature, °C	36.64 ± 2.18	36.56 ± 3.14	36.65 ± 2.09	36.71 ± 1.55	36.67 ± 1.40	0.620
Atrial fibrillation, n (%)						0.151
no	2308 (81.96)	608 (84.80)	647 (80.98)	487 (81.30)	566 (80.74)	
yes	508 (18.04)	109 (15.20)	152 (19.02)	112 (18.70)	135 (19.26)	
Respiratory failure, n (%)						<0.001
no	1891 (67.15)	474 (66.11)	575 (71.96)	401 (66.94)	441 (62.91)	
yes	925 (32.85)	243 (33.89)	224 (28.04)	198 (33.06)	260 (37.09)	
AKI, n (%)						0.041
no	687 (24.40)	170 (23.71)	215 (26.91)	123 (20.53)	179 (25.53)	
yes	2129 (75.60)	547 (76.29)	584 (73.09)	476 (79.47)	522 (74.47)	
Hypertension, n (%)						0.300
no	1880 (66.76)	499 (69.60)	527 (65.96)	390 (65.11)	464 (66.19)	
yes	936 (33.24)	218 (30.40)	272 (34.04)	209 (34.89)	237 (33.81)	
DM, n (%)						0.013
no	2006 (71.24)	540 (75.31)	575 (71.96)	408 (68.11)	483 (68.90)	
yes	810 (28.76)	177 (24.69)	224 (28.04)	191 (31.89)	218 (31.10)	
Heart failure, n (%)						0.083
no	2361 (83.84)	623 (86.89)	663 (82.98)	494 (82.47)	581 (82.88)	
yes	455 (16.16)	94 (13.11)	136 (17.02)	105 (17.53)	120 (17.12)	
MI, n (%)						0.638
no	2722 (96.66)	698 (97.35)	771 (96.50)	579 (96.66)	674 (96.15)	
yes	94 (3.34)	19 (2.65)	28 (3.50)	20 (3.34)	27 (3.85)	
Malignant tumors, n (%)						0.150
no	2559 (90.87)	664 (92.61)	729 (91.24)	534 (89.15)	632 (90.16)	
yes	257 (9.13)	53 (7.39)	70 (8.76)	65 (10.85)	69 (9.84)	
Sepsis, n (%)						<0.001
no	888 (31.53)	243 (33.89)	287 (35.92)	179 (29.88)	179 (25.53)	
yes	1928 (68.47)	474 (66.11)	512 (64.08)	420 (70.12)	522 (74.47)	
Liver transplantation, n (%)						0.027
no	2727 (96.84)	686 (95.68)	772 (96.62)	579 (96.66)	690 (98.43)	
yes	89 (3.16)	31 (4.32)	27 (3.38)	20 (3.34)	11 (1.57)	
180-day mortality, n (%)						<0.001
no	1758 (62.43)	462 (64.44)	535 (66.96)	379 (63.27)	382 (54.49)	
yes	1058 (37.57)	255 (35.56)	264 (33.04)	220 (36.73)	319 (45.51)	
365-day mortality, n (%)						<0.001
no	1593 (56.57)	423 (59.00)	490 (61.33)	337 (56.26)	343 (48.93)	
yes	1223 (43.43)	294 (41.00)	309 (38.67)	262 (43.74)	358 (51.07)	

Continuous variables are expressed as mean ± standard error, and categorical variables are expressed as numbers (%).

BCR, blood urea nitrogen to creatinine ratio; WBC, white blood cell; RBC, red blood cell; RDW, red blood cell distribution width; PT, prothrombin time; PTT, partial thromboplastin time; INR, international normalized ratio; ALT, alanine aminotransferase; AST, aspartate transaminase; SOFA, sequential organ failure assessment score; APS III, acute physiology score III; SIRS, systemic inflammatory response syndrome score; SAPS II, simplified acute physiology score II; OASIS, oxford acute severity of illness score; SBP, systolic blood pressure; DBP, diastolic blood pressure; MBP, mean blood pressure; SpO_2_, percutaneous arterial oxygen saturation; AKI, acute kidney injury; DM, diabetes mellitus; MI, myocardial infarction.

BCR quartiles: Q1 ≤ 14, 14<Q2 ≤ 20, 20<Q3 ≤ 27, and Q4>27.

### Association between BCR and mortality in critically ill cirrhosis patients

**Figure 2:**
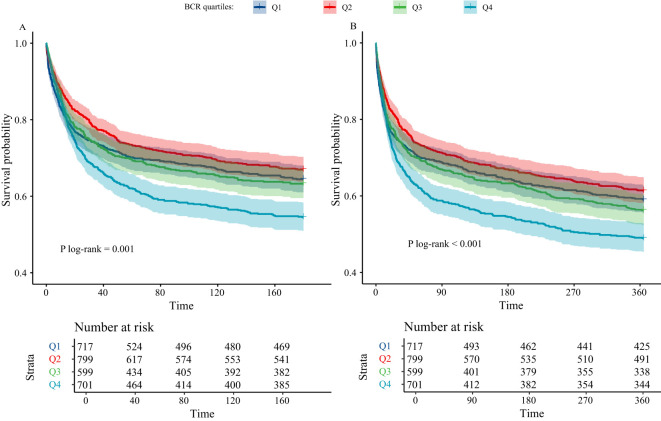
Kaplan-Meier analysis of critically ill cirrhotic patients according to BCR quartiles (**A**, 180-day mortality; **B**, 365-day mortality; BCR, blood urea nitrogen to creatinine ratio; BCR quartiles: Q1 ≤ 14, 14<Q2 ≤ 20, 20<Q3 ≤ 27, and Q4>27).

**Figure 2** presents the KM survival analysis evaluating the association between BCR and mortality in critically ill cirrhosis patients. **Figure 2A** illustrates the KM survival curve for 180-day mortality. The results showed that as the BCR quartile increased, the 180-day mortality of the patients significantly increased. Patients in the highest BCR quartile exhibited an increased mortality risk compared to those in the lowest quartile. This trend was further confirmed in the KM survival curve for 365-day mortality in **Figure 2B**. By Log-rank test, we found significant differences in the survival curves between different BCR quartiles (P < 0.05).

Our study investigated the association between BCR and all-cause mortality in critically ill cirrhosis patients using a multivariate Cox regression model (**Table 2**). The results showed that the BCR ratio was significantly associated with both 180-day and 365-day mortality. In Model 1, without corrections, For every 1-unit rise in BCR, there was a corresponding 1% increase in the risk of death at both 180 and 365 days (P<0.001). Even after adjusting for additional confounders in Model 3, the association between BCR and mortality persisted significantly (P<0.001).

**Table 2:** The association between BCR and all-cause mortality in critically ill patients with cirrhosis.

	Model 1		Model 2		Model 3	
	HR (95%CI)	*P*	HR (95%CI)	*P*	HR (95%CI)	*P*
180-day mortality						
BCR	1.01 (1.01,1.02)	<0.001	1.01 (1.01,1.02)	<0.001	1.01 (1.01,1.02)	<0.001
BCR quartiles						
Q1	ref		ref		ref	
Q2	0.89 (0.75,1.05)	0.175	0.86 (0.72,1.02)	0.078	1.06 (0.89,1.26)	0.523
Q3	1.02 (0.85,1.22)	0.822	0.97 (0.81,1.16)	0.737	1.06 (0.88,1.28)	0.558
Q4	1.33 (1.13,1.57)	<0.001	1.28 (1.08,1.51)	0.004	1.45 (1.21,1.73)	<0.001
P for trend	0.008		0.009		<0.001	
365-day mortality						
BCR	1.01 (1.01,1.02)	<0.001	1.01 (1.01,1.02)	<0.001	1.01 (1.01,1.02)	<0.001
BCR quartiles						
Q1	ref		ref		ref	
Q2	0.90 (0.77,1.06)	0.205	0.87 (0.74,1.02)	0.085	1.04 (0.90,1.23)	0.598
Q3	1.06 (0.90,1.25)	0.487	1.01 (0.85,1.19)	0.944	1.08 (0.91,1.28)	0.391
Q4	1.32 (1.13,1.54)	<0.001	1.27 (1.09,1.48)	0.003	1.38 (1.17,1.63)	<0.001
P for trend	0.020		0.023		<0.001	

Model 1: no adjustment.

Model 2: adjust for age and weight.

Model 3: adjust for age, weight, WBC, RDW, hemoglobin, potassium, chloride, glucose, anion gap, INR, AST, SOFA, APS III, SIRS, heart rate, AKI, diabetes, sepsis, and liver transplantation.

BCR, blood urea nitrogen to creatinine ratio; WBC, white blood cell; RDW, red blood cell distribution width; INR, international normalized ratio; AST, aspartate transaminase; SOFA, sequential organ failure assessment score; APS III, acute physiology score III; SIRS, systemic inflammatory response syndrome score; AKI, acute kidney injury; HR, hazard ratio; CI, confidence interval.

BCR quartiles: Q1 ≤ 14, 14<Q2 ≤ 20, 20<Q3 ≤ 27, and Q4>27.

For BCR quartiles, the highest quartile (Q4) showed a significant increase in both 180-day and 365-day mortality compared with the lowest quartile (Q1). Model 3 demonstrated a heightened risk of mortality, with a 45% increase for 180-day mortality (HR=1.45, 95% CI: 1.21-1.73) and a 38% increase for 365-day mortality (HR=1.38, 95% CI: 1.17-1.63). In addition, the association between different BCR quartiles and mortality was also differential (P for trend <0.001).

### A linear association

**Figure 3:**
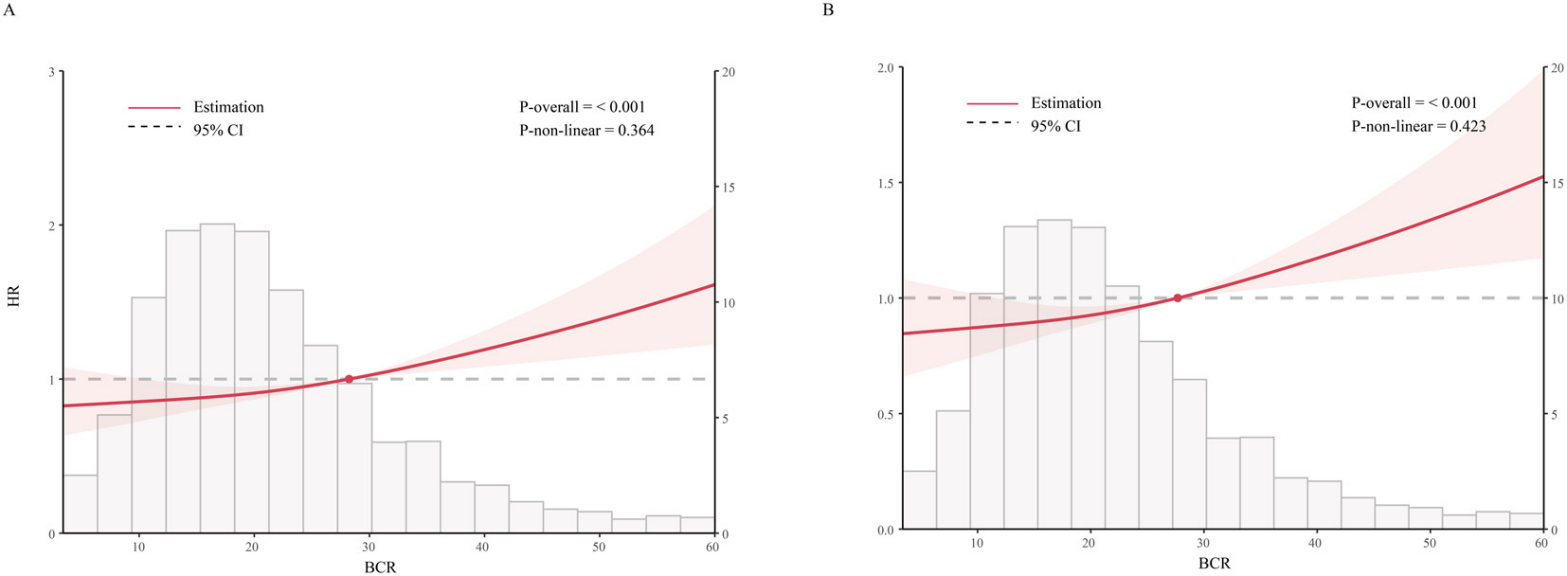
Restricted cubic spline analysis of mortality risk with BCR in critically ill patients with cirrhosis (**A**, 180-day mortality; **B**, 365-day mortality; BCR, blood urea nitrogen to creatinine ratio).

**Figure 3** presents an RCS analysis examining the association between BCR and mortality risk at 180 days (**Figure 3A**) and 365 days (**Figure 3B**) in critically ill cirrhosis patients. The RCS curves indicate a significant linear correlation between BCR and 180-day mortality risk (nonlinear P = 0.364), with increased BCR values associated with a higher risk of death. The 180-day risk of death in cirrhotic patients increased with higher BCR values. Similarly, a similar linear association was found in the 365-day mortality study (nonlinear P = 0.423).

### Subgroup analysis

**Figure 4:**
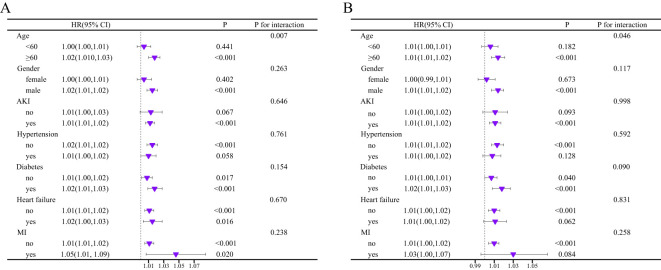
Subgroup analysis of the association between BCR and mortality risk of critically ill cirrhosis (**A**, 180-day mortality; **B**, 365-day mortality).

We employed a forest plot to illustrate the relationship between BCR and mortality at 180 and 365 days in critically ill cirrhosis patients with varying clinical characteristics (**Figure 4**). Specifically, positive associations between BCR and all-cause mortality were shown in patients of different ages, gender, AKI, hypertension, DM, heart failure, and myocardial infarction. The interaction test results indicated a notable variance in the relationship between BCR and mortality among critically ill cirrhosis patients across different age subgroups. The positive correlations between BCR and mortality at 180 and 365 days were more pronounced in patients over 60 compared to those under 60. In other subgroups, no significant interactions were found.

## Discussion

This study explored the association between BCR and mortality in critically ill cirrhosis patients. Our analysis of MIMIC-IV data indicates a strong link between high BCR and higher mortality risk in patients. KM survival analysis and multivariate Cox regression models consistently showed that elevated BCR quartiles significantly increased mortality at both 180 and 365 days. RCS analysis demonstrated a significant linear correlation between increased BCR values and elevated mortality risk. Subgroup analyses and interaction effects revealed a stronger association between BCR and mortality in older patients.

BCR, as a simple biochemical marker, has gained increasing recognition for its prognostic significance in various clinical settings. Previous research has demonstrated a significant association with negative clinical outcomes, such as acute kidney injury, heart failure, cerebral infarction, and ischemic stroke (29–33). A prospective cohort study of over 50,000 participants, with an average follow-up of 7.9 years, found that elevated BCR levels were linked to a higher stroke risk (19). Additionally, BCR is linked to a heightened risk of insulin resistance (34). The uniform association between BCR and mortality across various studies underscores the significance of this marker in forecasting patient outcomes. In patients with chronic heart failure, Paolo et al. found that a higher BCR was associated with adverse outcomes in chronic heart failure patients, independent of estimated glomerular filtration rate (eGFR) and N-terminal pro-brain natriuretic peptide (NT-proBNP), and may be associated with pathophysiological mechanisms such as neurohormonal activation and changes in renal blood flow (18). Our study supports the role of BCR as a prognostic indicator in cirrhosis patients, aligning with previous findings.

The relationship between BCR and mortality in cirrhotic patients involves complex mechanisms. BCR reflects the kidney’s ability to excrete urea and creatinine, and elevated levels may indicate impaired renal function, a known predictor of poor outcomes in cirrhosis (17, 35). In addition, extreme impairment of renal function in patients with cirrhosis leads to hepatorenal syndrome, which is characterized by a decrease in renal blood flow and glomerular filtration rate, which also leads to an elevated BCR, which likewise increases the poor prognosis of patients with cirrhosis (36).

The subgroup analysis identified a notable interaction between age and BCR as predictors of mortality. Older patients with higher BCR levels exhibited a more pronounced increase in mortality risk. This interaction might result from age-related renal function decline, which is associated with higher BCR values. Elevated BCR may indicate a poorer prognosis in older patients with cirrhosis (37). Additionally, older patients often have a higher burden of comorbidities that, in conjunction with elevated BCR, contribute to a higher risk of mortality (38). This finding emphasizes the importance of considering age as a modifier when evaluating the prognostic value of BCR in cirrhotic patients.

Our study highlights the potential of blood urea nitrogen to creatinine ratio (BCR) as a prognostic marker for patients with cirrhosis. The BCR has several advantages over existing prognostic scores such as the Child-Pugh score and the MELD score. It is a direct biochemical indicator of both renal and hepatic function. In addition, the BCR avoids the subjectivity of certain markers (e.g., ascites and encephalopathy) in the Child-Pugh score and the laboratory heterogeneity of creatinine and INR measurements in the MELD score, as well as gender bias (39–41). Our findings suggest that the BCR can be a valuable adjunct to existing prognostic tools, especially in critically ill patients where an accurate prognosis is essential for clinical decision-making.

While our study provides valuable insights into predicting mortality in critically ill cirrhotic patients, it has limitations. Firstly, our analysis is retrospective, which limits the ability to establish causality and may introduce biases inherent in observational studies. Secondly, the single-center origin of our patient cohort may restrict the applicability of our findings to diverse populations and healthcare environments. Thirdly, despite adjusting for many confounders, some unincluded variables such as laboratory tests, genetic factors, lifestyle, and specific treatments may still affect the relationship between BCR and mortality, and thus the scope of attention in the interpretation of the conclusions. Fourth, due to missing data from retrospective studies, we were unable to obtain all the metrics for either the MELD score or the ACLF score, so they were not included in the analyses, and we will take these two scores into account in future prospective studies. Our study adds to the increasing evidence highlighting the significance of BCR in managing critically ill cirrhotic patients, despite certain limitations.

## Conclusion

In cirrhotic patients admitted to the ICU, a higher BCR is associated with increased short- and long-term mortality. Therefore, the measurement of BCR may be helpful in the prognostic management of cirrhotic patients in the ICU. Additional prospective studies are required to confirm our results.

## Data Availability

The raw data supporting the conclusions of this article will be made available by the authors, without undue reservation.
