# Supplementary material for: Interaction between age and blood urea nitrogen to creatinine ratio on mortality in patients with severe cirrhosis: a retrospective cohort study from the MIMIC database
Source: Front Endocrinol (Lausanne). 2025 Mar 5;16:1544223. doi: 10.3389/fendo.2025.1544223 (PMC11919653; doi:10.3389/fendo.2025.1544223)
Supplement: Supplementary file 1 [file DataSheet1.docx]

**Supplementary Material**

Supplementary Table 1. List of missing variables and their missingness rates.

Supplementary Figure 1. Schoenfeld residual plots.

| Supplementary Table 1. List of missing variables and their missingness rates | | |
| --- | --- | --- |
| **Variables** | **Missing (n)** | **Complete rate (%)** |
| Age | 0 | 1.000 |
| Gender | 0 | 1.000 |
| Weight | 44 | 0.984 |
| Atrial fibrillation | 0 | 1.000 |
| Respiratory failure | 0 | 1.000 |
| AKI | 0 | 1.000 |
| Hypertension | 0 | 1.000 |
| DM | 0 | 1.000 |
| Heart failure | 0 | 1.000 |
| MI | 0 | 1.000 |
| Malignant tumors | 0 | 1.000 |
| Sepsis | 0 | 1.000 |
| Liver transplantation | 0 | 1.000 |
| WBC | 10 | 0.996 |
| RBC | 10 | 0.996 |
| Neutrophil | 2059 | 0.273 |
| Lymphocyte | 2059 | 0.273 |
| Platelet | 17 | 0.994 |
| Hemoglobin | 12 | 0.996 |
| RDW | 11 | 0.996 |
| Albumin | 474 | 0.833 |
| Sodium | 0 | 1.000 |
| Potassium | 1 | 1.000 |
| Calcium | 15 | 0.995 |
| Chloride | 0 | 1.000 |
| Glucose | 0 | 1.000 |
| HBA1c | 2587 | 0.087 |
| Anion gap | 0 | 1.000 |
| Lactic acid | 697 | 0.754 |
| Thrombin time | 2799 | 0.012 |
| PT | 71 | 0.975 |
| Fibrinogen | 1385 | 0.511 |
| PTT | 87 | 0.969 |
| INR | 72 | 0.975 |
| Triglyceride | 2255 | 0.204 |
| Total cholesterol | 2591 | 0.085 |
| High-density lipoprotein | 2629 | 0.072 |
| Low-density lipoprotein | 2635 | 0.070 |
| ALT, u/dL | 182 | 0.936 |
| AST, u/dL | 169 | 0.940 |
| Blood urea nitrogen | 0 | 1.000 |
| Creatinine | 0 | 1.000 |
| Uric acid | 2727 | 0.037 |
| SOFA | 0 | 1.000 |
| APS III | 0 | 1.000 |
| SIRS | 0 | 1.000 |
| SAPS II | 0 | 1.000 |
| OASIS | 0 | 1.000 |
| Heart rate | 0 | 1.000 |
| SBP | 40 | 0.986 |
| DBP | 40 | 0.986 |
| MBP | 40 | 0.986 |
| Heart rate | 3 | 0.999 |
| SpO2 | 3 | 0.999 |
| Temperature | 43 | 0.985 |
| BCR | 0 | 1.000 |
| BCR, blood urea nitrogen to creatinine ratio; WBC, white blood cell; RBC, red blood cell; RDW, red blood cell distribution width; PT, prothrombin time; PTT, partial thromboplastin time; INR, international normalized ratio; ALT, alanine aminotransferase; AST, aspartate transaminase; SOFA, sequential organ failure assessment score; APS III, acute physiology score III; SIRS, systemic inflammatory response syndrome score; SAPS II, simplified acute physiology score II; OASIS, oxford acute severity of illness score; SBP, systolic blood pressure; DBP, diastolic blood pressure; MBP, mean blood pressure; SpO_2_, percutaneous arterial oxygen saturation; AKI, acute kidney injury; DM, diabetes mellitus; MI, myocardial infarction. | | |


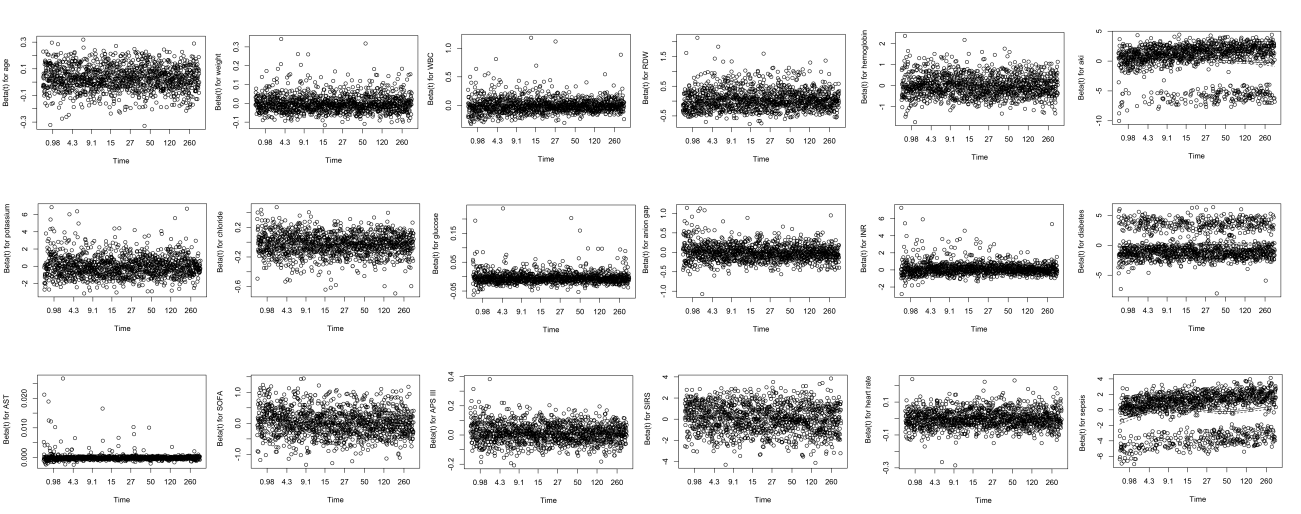


Supplementary Figure 1. Schoenfeld residual plots.
